# Seroprevalence of human T lymphotropic virus antibodies among healthy blood donors at a tertiary centre in Lagos, Nigeria

**DOI:** 10.11604/pamj.2014.17.301.4075

**Published:** 2014-04-21

**Authors:** Idris Durojaiye, Akinsegun Akinbami, Adedoyin Dosunmu, Sarah Ajibola, Adewumi Adediran, Ebele Uche, Olajumoke Oshinaike, Majeed Odesanya, Akinola Dada, Olaitan Okunoye

**Affiliations:** 1 Department of Haematology and Blood Transfusion Lagos State University, Teaching Hospital, Lagos, Nigeria; 2 Department of Haematology and Blood Transfusion, Lagos State University, College of Medicine, ikeja, Nigeria; 3 Department of Haematology and Immunology, Ben Carson, School of Medicine, Backcok University, Ilisan, Ogun State, Nigeria; 4 Department of Haematology and Blood Transfusion, Faculty of Clinical Sciences, College of Medicine, University of Lagos, Lagos, Nigeria; 5 Department of Medicine, Lagos State University, College of Medicine, Ikeja, Nigeria; 6 Oak Hospitals, Ikorodu, Nigeria; 7 Department of Medicine, University of PortHarcourt, River State, Nigeria

**Keywords:** Seroprevalence, HTLV, healthy blood donors, Nigeria

## Abstract

**Introduction:**

Transmission of human T-lymphotropic viruses (HTLV) occurs from mother to child, by sexual contact and blood transfusion. Presently, in most centres in Nigeria, there is no routine pre-transfusion screening for HTLV. The study aims to determine the prevalence of HTLV-1 and HTLV-2 among healthy blood donors at a tertiary centre in Lagos.

**Methods:**

A cross-sectional study was carried out at the blood donor clinic of the Lagos State University Teaching Hospital (LASUTH), Ikeja. About 5mls of venous blood was collected from each subject into a sterile plain bottle after obtaining subject's consent. The serum separated and stored at -200C. Sera were assayed for HTLV by an enzyme-linked immunoassay (ELISA) for the determination of antibodies to HTLV 1 and HTLV -2. Western blot confirmatory testing was done on reactive samples. All donors were also screened for HIV, HBsAg and HCV by rapid kits.

**Results:**

The seroprevalence of HTLV -1 by ELISA was 1.0% and 0.5% by Western Blot among blood donors. A total of 210 healthy blood donors were enrolled. Only 2 (1.0%) blood donors were repeatedly reactive with ELISA test. On confirmatory testing with Western Blot, 1 (0.5%) blood donor was positive for HTLV. All the healthy blood donors were negative for HIV, HbsAg and HCV. None of the 210 blood donors had been previously transfused; as such no association could be established between transfusion history and HTLV positivity among the blood donors.

**Conclusion:**

The seroprevalence of HTLV in this environment is low among healthy blood donors.

## Introduction

The human T-lymphotropic viruses, type 1 (HTLV-1) and type 2 (HTLV-2), were the first human retroviruses discovered [1, 2]. They are single-stranded RNA retroviruses of the so-called C type originally described by Gallo′s group at the National Cancer Institute in 1980 and 1982, respectively [2, 3]. Human T cell leukaemia/lymphoma virus type I (HTLV-1), the first human oncoretrovirus to be discovered [1], causes a lymphoproliferative malignancy of CD4-activated cells called adult T-cell leukaemia/lymphoma (ATL) and a chronic myelopathy called tropical spastic paraparesis/HTLV-1-associated myelopathy (TSP/HAM) [4]. HTLV-2 has a similar genome structure and shares approximately 70% nucleotide sequence homology with HTLV-1. [5] There is also significant association of HTLV-1 with lymphoid malignancies [6].

Infections of HTLV-1 and HTLV-2 are lifelong with asymptomatic carrier state [3]. Over 20 million persons are infected with HTLV-1and HTLV-2 globally with varying levels of seroprevalence reported in almost every region of the world [7]. These retroviruses are found in foci of micro-endemicity, particularly in southern Japan [8], equatorial Africa [9, 10], and parts of the Americas, including the Caribbean basin [11], and the South-Eastern USA [11]. The frequency of antibodies in symptom-free adults throughout Sub-Saharan Africa has been reported to be from 3 to 4% [12, 13].

Transmission of HTLV-1 occurs from mother to child [14, 15], by sexual contact [16], by blood transfusion [17, 18], and by sharing contaminated needles [17, 19]. Mother-to-child transmission occurs primarily by breast-feeding through ingestion of infected milk-borne lymphocytes [20]. In HTLV-1-endemic areas, approximately 25% of breast-fed infants born to HTLV-1-seropositive mothers acquire infection [20]. The transmission efficiency is dependent on the duration of breastfeeding and the presence of maternal antibodies to HTLV-1 [21, 22]. The time of infant seroconversion typically ranges from 1 to 3 years of age [20, 22]. Intrauterine or perinatal transmission of HTLV-1 does occur, but it appears to be less frequent than transmission by breast-feeding; approximately 5% of children born to infected mothers but not breast-fed acquire infection [21].

Sexual transmission of HTLV-1 is bi-directional [16, 23]. However, the frequency of HTLV-1 transmission is much higher from male to female than from female to male [23, 24]. The presence of genital ulcers increases the risk of virus transmission [24].

Transmission of HTLV-1 by blood transfusion occurs with transfusion of cellular blood products (whole blood, red blood cells, and platelets) but not with the plasma fraction or plasma derivatives from HTLV-1-infected blood [18]. Seroconversion rates of 44% to 63% have been reported in recipients of HTLV-1-infected cellular components in HTLV-1 endemic areas [17, 18]. The probability of transmission by whole blood or packed red blood cells appears to diminish with greater duration of product storage; this finding has been ascribed to depletion of infected cells, presumably T-lymphocytes [18, 25]. Sharing blood-contaminated needles is the likely mode of transmission among intravenous drug users (IDUs) [26].

As HTLVs are transmitted through blood transfusion, screening for antibodies and discarding seropositive units should efficiently interrupt this transmission. Concern about HTLV-1 transmission through blood transfusion has led to the introduction of routine blood-donor screening for antibodies to HTLV-1 in developed countries [27, 28]. The decision to extend universal screening of blood donations to all industrialised countries with a low prevalence is a matter of debate and it has been suggested that the decision should be made country by country [29].

Presently at the Lagos State University Teaching Hospital (LASUTH), as in most other centres in Nigeria, there is no routine pre-transfusion screening for HTLV-1. The study aims to determine the prevalence of HTLV-1and HTLV- 2 among healthy blood donors at a tertiary centre in Lagos.

## Methods

A cross-sectional study was carried out at the blood donor clinic of the Lagos State University Teaching Hospital (LASUTH), Ikeja, between February and May 2012.

### Erhical considerations and clearance

Ethical clearance was obtained from the Health Research and Ethics Committee of LASUTH.

### Inclusion and exclusion criteria for blood donors

**Inclusion criteria:** All fit blood donors who gave informed consent during the period of the study.

**Exclusion criteria:** Individuals with risk factors for Transfusion Transmissible Infections (TTI). 2. Individuals who were positive for HIV and/or hepatitis B on rapid screening. 3. Donors who did not give consent.

### Sample size determination

Sample size was determined using the Yamane (Sloven) [29] formula:

N = N/ (1 + N (e) 2)

Where: n = minimum sample size required; N = the population size; e = degree of accuracy desired i.e. confidence interval, expressed as a decimal (±5% = ±0.05)

### Minimum sample size for healthy blood donors

From LASUTH Blood Bank, records of average number of healthy blood donors seen over a three month period;

N = 400 n =400/ (1 + 400(0.05)2) =400/ (1 + 400(0.0025)) = 400/1 + 1 = 400/2 n= 200; Minimum sample size = 200 healthy blood donors.

### Specimen collection and storage

About 5mls of venous blood was collected from each subject into a sterile plain bottle after obtaining subject's consent. The blood was allowed to clot completely before centrifugation. The serum was separated within 6 hours and stored in sterile cryovials at -200C.

### Sample assay

Serum samples were assayed for HTLV using the HTLV 1 and 2 Ab versions ULTRA by Diagnostic Bioprobes Srl, Italy, an enzyme-linked immunoassay for the determination of antibodies to HTLV 1 and HTLV-2 in serum and plasma. Western blot confirmatory testing (MP Diagnostics (MPD) HTLV Blot 24 Singapore) was done on reactive samples.

### Screening for other transfusion transmissible infections among donors

The sera of donors were also tested for the presence of antibodies to the human immunodeficiency virus (HIV) with Alere Determine TM HIV-1/2 test kit, ( Belgium) hepatitis B surface antigen (HbsAg) with Micropoint HbsAg Gold TM rapid screen test (Trinity Biotech Plc, Japan) and hepatitis C virus with DiaSpot HCV test kit (Sam Tech Diagnostic, China). They are rapid screening test kits for the qualitative detection of the respective antibodies in whole blood, plasma or serum.

### Data analysis and presentation

The data was recorded in compatible computer and analysed with

**Epi-Info version 3.5.3 software**. The mean, median, standard deviation and other parameters of statistical location were generated as necessary for continuous data. Tests of statistical significance between variables included chi-square analysis and Fischer's exact for discrete data. Level of significance was set at p < 0.05.

## Results

The seroprevalence of HTLV -1 by ELISA was 1.0% and 0.5% by Western Blot among blood donors. None of the 210 blood donors had been previously transfused; as such no association can be established between transfusion history and HTLV-1 positivity among the blood donors.

A total of 210 healthy blood donors were enrolled. Of the healthy blood donors, 184 (87.6%) were males and 26 (12.4%) were females (Table 1). The mean age of the overall healthy blood donors’ was 33 ± 8.9 years, male blood donors 32.8 ± 8.5 years and of female blood donors was 34.6 ± 11.3 years. The mean age of male blood donors 32.8 ± 8.5 years was not significantly different from that of the female donors 34.6 ± 11.3 years. Of the 210 healthy blood donors, 93 (44.3%) were single and 117 (55.7%) were married (Table 2). Of the healthy blood donors, 66 (31.4%) had a maximum of secondary education, while among the patients 19 (48.7%) had a maximum of secondary education. Among the blood donors 144 (68.6%) had at least post-secondary education, while 20 (51.3%) of the patients had at least tertiary education.

**Table 1:** Age and sex distribution of subjects

	Healthy blood donors		
Age In Years	Male	Female	Total
<20	5	-	5
20- 29	67	12	79
30 -39	75	6	81
40 – 49	29	5	34
51 – 59	8	3	11
60 – 69	-	-	-
70 – 79	-	-	-
80 – 89	-	-	-
90 – 99	-	-	-
**Total (%)**	**184 (87.6)**	**26 (12.4)**	**210 (100.0)**

**Table 2:** Marital status of the subjects

Marital status	Healthy donors	%
Single	93	44.3
Married	117	55.7
Divorced/separated	-	-
Widowed	-	-
**Total**	**210**	**100.0**

All the 93 single blood donors were HTLV-1 negative. Of the 117 married blood donors, 1 (0.9%) was HTLV-1 positive and 116 (99.1%) were HTLV-1 negative. There was no association between marital status and HTLV-1 positivity. P = 0.324 Of the 210 blood donors, only 1 (0.5%) was HTLV-1 positive and was aged 23 years (age range 20 -29 years). P = 0.614. Among the 184 male healthy blood donors, only 1(0.5%) was HTLV-1 positive. All 26 females were HTLV-1 negative. P = 1.000. Only 2 (1.0%) blood donors were repeatedly reactive with ELISA test. On confirmatory testing with Western Blot, 1 (0.5%) blood donor was positive for HTLV-1.(Table 3) All the healthy blood donors were negative for HIV, HbsAg and HCV.

**Table 3:** HTLV-1 status of the subjects

HTLV-1	Healthy donors	%
Negative	209	99.5
Positive	1	0.5
**Total**	**210**	**100.0**

## Discussion

The seroprevalence of HTLV-1 of 0.5% among healthy blood donors in this study is similar to the study by Analo et al in 1998 in Lagos in which 0.7% of blood donors were HTLV-1 positive [31]. It is lower than that from adult blood donors in Lagos (1.8%) in a 1986 study by Fleming et al [32], and also lower than average nationwide rates for blood donors in Nigeria (2 to 4.8%) [13]. This may be due to stricter donor selection criteria currently in place since the advent of HIV/AIDS and use of newer highly specific test kits with fewer false positives in the present study.

The prevalence of HTLV-1 among blood donors in this study is however much higher than that among blood donors in North America and Europe, where seroprevalence is very low, for example, 0.01-0.03% in USA and Canada [33, 34], 0.0039 in France [34], 0.002% in Norway [27] and 0.0056% in Greece [36]. Stricter donor selection criteria may account for the very low HTLV-1 seroprevalence rates in these countries [27, 28].

History of previous blood transfusion appeared to have no impact on HTLV-1 status in this study. None of the blood donors had been previously transfused. This is at variance to studies that reported past history of transfusion as an important risk factor for HTLV-1 seropositivity [37], Several studies have demonstrated higher HTLV-1 prevalence with increasing age [38, 39] Conversely, the only blood donor who was HTLV-1 positive was below 30 years of age.

This is not in conformity with studies that have shown the frequency of HTLV-1 transmission to be much higher from male to female than from female to male [23, 24]. This is most likely due to the higher proportion of males compared with females in this study. This is another limitation of the study as gender of the participants was skewed in favour of the male sex. However, sexual difference was not statistically significant in this study. P = 0.490

## Conclusion

The seroprevalence of HTLV-1 in this environment is low among healthy blood donors.
